# Perspectives of Community Health Physicians on Access to Neurosurgical Care in the Philippines

**DOI:** 10.7759/cureus.103661

**Published:** 2026-02-15

**Authors:** Christopher Brian M Reyes, Rhoby U Orata, Abdul A Ontok

**Affiliations:** 1 Neurosurgery, Armed Forces of the Philippines Health Service Command, V. Luna Medical Center, Quezon City, PHL; 2 Surgery, Armed Forces of the Philippines Health Service Command, V. Luna Medical Center, Quezon City, PHL

**Keywords:** community health, neurosurgery, neurosurgical access, neurosurgical care, rural neurosurgery

## Abstract

Access to essential neurosurgical care in low and middle-income countries (LMICs), particularly in the Philippines, is a significant challenge, exacerbated by financial, logistical, and staffing barriers. Despite a high burden of neurological disorders, access to neurosurgical services remains limited in many areas. This cross-sectional study employed a survey targeting community health physicians across the Philippines, utilizing snowball sampling to ensure diverse representation. A structured questionnaire gathered data on the availability of neurosurgical services, referral practices, and perceived barriers to access, focusing on geographical, economic, and systemic challenges. Valid responses were collected from 63 participants, primarily public health officers. The data revealed that a significant number of physicians encounter neurosurgical cases monthly, predominantly strokes and traumatic brain injuries. Participants from the Visayas and Mindanao Islands reported more frequent consultations, occurring on a daily to weekly basis. However, logistical challenges such as long referral distances and inadequate local resources were prevalent, with many communities lacking access to essential diagnostic tools. Furthermore, a majority of participants reported the absence of a structured follow-up system for postoperative care. The findings highlight urgent barriers to accessing neurosurgical care, including financial constraints and geographical isolation. Participants emphasized the need for improved referral systems, increased local government support, and enhanced training for healthcare providers. This study underscores the critical need for comprehensive strategies to enhance access to neurosurgical services in the Philippines, focusing on infrastructural improvements, community awareness, and resource allocation to reduce morbidity associated with delayed treatment in underserved regions.

## Introduction

Access to essential health care services was a significant challenge in low and middle-income countries (LMICs). Approximately two billion people worldwide lacked access to basic surgical care [1]. Barriers such as financial constraints, logistical issues, and inadequate staffing and training worsened this problem. Access to speciality services, including neurosurgery, was even more restricted. For example, an estimated 4.5 billion people globally did not have access to cardiac surgery, reflecting a stark disparity between high and low-income regions [2]. Similar gaps in access to essential surgical and neurosurgical services have been reported across LMICs, where resource limitations, geographic isolation, and uneven workforce distribution continue to challenge health systems [3].

Each year, around 22.6 million people suffer from neurological disorders or injuries requiring neurosurgical expertise, with 13.8 million needing surgical intervention [3]. Inadequate neurosurgical care could lead to increased patient mortality and morbidity due to complications from delayed or insufficient treatment. Despite being one of the first countries to establish neurosurgical units in Southeast Asia, the Philippines faced momentous challenges. With a population of 100 million and only 120 neurosurgeons, there was only one neurosurgeon per 807,000 people [4,5]. This proportion contrasts sharply with other first-world countries like Japan, which had over 7,500 neurosurgeons, including 5,432 board-certified specialists [6].

There was currently no national census or database of all neurosurgical cases in the Philippines. Estimates suggested approximately 3,504,783 neurosurgical cases in Southeast Asia and 13,786,823 worldwide [7]. Thirty-five Philippine provinces had no neurosurgeons at all, which corresponded to an approximately 24 million population [6]. This shortage of the neurosurgical workforce opens up the disparity of the level of access to neurosurgical care across different setting which could mostly be driven by the financial capacity of the various locales.

Given these challenges, understanding access to neurosurgical care from the perspective of frontline providers is critical. The primary objective of this study was to describe access to neurosurgical care in the Philippines from the perspectives of community health physicians. The secondary objectives were to characterize referral practices, identify commonly encountered neurosurgical conditions, and explore perceived barriers to accessing neurosurgical services at the community level. This study was designed as a descriptive and exploratory investigation to generate context-specific insights that may inform health policy, workforce planning, and system-level interventions aimed at improving neurosurgical care access in resource-limited settings.

## Materials and methods

Study design

The study employed a prospective, cross-sectional design to evaluate access to neurosurgical care from the viewpoint of community health physicians across the Philippines. The study was descriptive and exploratory in nature, designed to characterize access, referral practices, and perceived barriers rather than to establish causal relationships. The primary objective is to gather quantitative data on the availability and accessibility of neurosurgical services, as well as to identify perceived barriers to care. The survey was conducted from September 15, 2024, to October 30, 2024.

Participants

Participants in this study were community health physicians from across the Philippines, selected through snowball sampling and included all eligible participants during the data collection period to ensure diverse representation from all island groups in the Philippines, as well as various community settings.

The inclusion criteria for participants required them to be community health physicians who were currently practising or had practised within the last 10 years, with at least one year of experience in their roles. A minimum of one year of experience was required to ensure sufficient familiarity with local healthcare systems, referral pathways, and community-level barriers to accessing specialized care. Additionally, participants had to be involved in patient referrals or care management within their communities, as their perspectives were crucial for evaluating access and identifying barriers to neurosurgical services.

The exclusion criteria included community health physicians with less than one year of experience and those not involved in patient referral processes. Furthermore, physicians who worked exclusively in private healthcare settings or had limited their practice to hospital environments were excluded to maintain a deliberate focus on the public and community health system, where access constraints to neurosurgical services are most pronounced. By establishing these criteria, the study aimed to collect relevant and meaningful data from individuals who were most familiar with the issues surrounding neurosurgical access in the Philippines.

Participants were invited through email to personal contacts and social media platforms, with referrals facilitated through these contacts (WhatsApp and Viber), forming a snowball sampling referral system. Snowball sampling was selected due to the geographically dispersed nature of community health physicians across the Philippine archipelago and the absence of a centralized registry of such practitioners. To promote geographic diversity, initial contacts were drawn from different island groups, and participants were encouraged to refer colleagues practising in geographically isolated and underserved areas, including geographically isolated and depressed areas (GIDAs). The survey was self-reporting and may be a source of bias in the study.

Setting and data collection

Data collection involved a structured questionnaire designed to assess several key variables related to neurosurgical care access. These variables included the availability of neurosurgical services, referral patterns, and obstacles faced in accessing care. Additionally, the survey gathered demographic information and contextual factors that may have influenced perceptions of care access. To ensure participant confidentiality, responses were anonymized, and participants were categorized by region, the presence of GIDAs in their areas, and the class of municipality (ranging from first to sixth class).

The survey was administered electronically via Google Forms, featuring a semi-structured questionnaire that covered essential topics such as the management of neurosurgical cases, protocols and procedures followed, and referral practices for directing patients to secondary or tertiary hospitals. It also examined the distance of referral hospitals from GIDA communities, the availability and effectiveness of local government support in terms of health financing and logistics, follow-up care systems for monitoring neurosurgical patients post-treatment, and various barriers to care, including geographic, cultural, technical, logistical, and technological obstacles (Appendix A). To eliminate bias, the analysis of responses was conducted by authors who were not involved in the survey's design.

Operational definitions

For this study, a community health physician was defined as a licensed medical doctor practising in a public primary care, rural health unit, municipal health office, or similar community-based setting, with direct involvement in patient care coordination and referral processes. These physicians serve as frontline providers within the Philippine public health system and are distinct from hospital-based specialists or private practitioners.

Primary hospitals were defined as facilities providing basic emergency and inpatient services without on-site neurosurgical capability. Secondary hospitals referred to facilities with broader diagnostic and surgical services but without full-time neurosurgical coverage. Tertiary hospitals were defined as referral centers with specialised services, including dedicated neurosurgical units. Neurosurgical capability, as used in this study, referred to the availability of on-site neurosurgeons and the capacity to perform definitive neurosurgical procedures, rather than the ability to provide initial stabilization or referral. GIDAs were treated as geographic classifications based on physical isolation, limited infrastructure, or socioeconomic disadvantage and were not used to characterize physician roles or competencies.

Statistical analysis

Descriptive analysis was conducted to provide an overview of the demographic characteristics of the participants, including years of experience, previous or current employment as a community health physician and type of practice setting (rural vs. urban and first class vs sixth class). Frequency distributions and percentages were used to present categorical variables, such as the availability of resuscitative services and referral practices. For continuous variables, measures such as means, medians, and standard deviations were calculated to summarize the data on aspects like the distance of referral hospitals and the presence of GIDAs in their area of practice. Additionally, descriptive statistics were employed to assess participants' perceptions regarding barriers to accessing neurosurgical care. This included summarizing responses related to geographic, cultural, technical, logistical, and technological challenges. Data visualizations, such as charts and graphs, were created to facilitate the interpretation of findings and highlight key trends in the data.

## Results

In total, the study garnered 71 responses. Two responses were removed for being duplicates, and six responses were deleted as they were not from community health physicians but physicians from primary hospitals in GIDAs. The study gathered valid responses from 63 community health physicians across the Philippines, and all valid respondents completed the survey. Among the participants, 37 (59%) are currently serving as community health physicians, while 26 (41%) have transitioned to different practices after working as community health physicians. Most of these physicians previously worked in the Doctors to the Barrios program, which deploys medical personnel to underserved areas. Some participants chose not to take part in the Department of Health’s absorption program, which offers a pathway to permanent employment in their chosen communities. However, 46 (74.2%) of those who were previously community health physicians have worked in their communities within the past five years.

**Figure 1:**
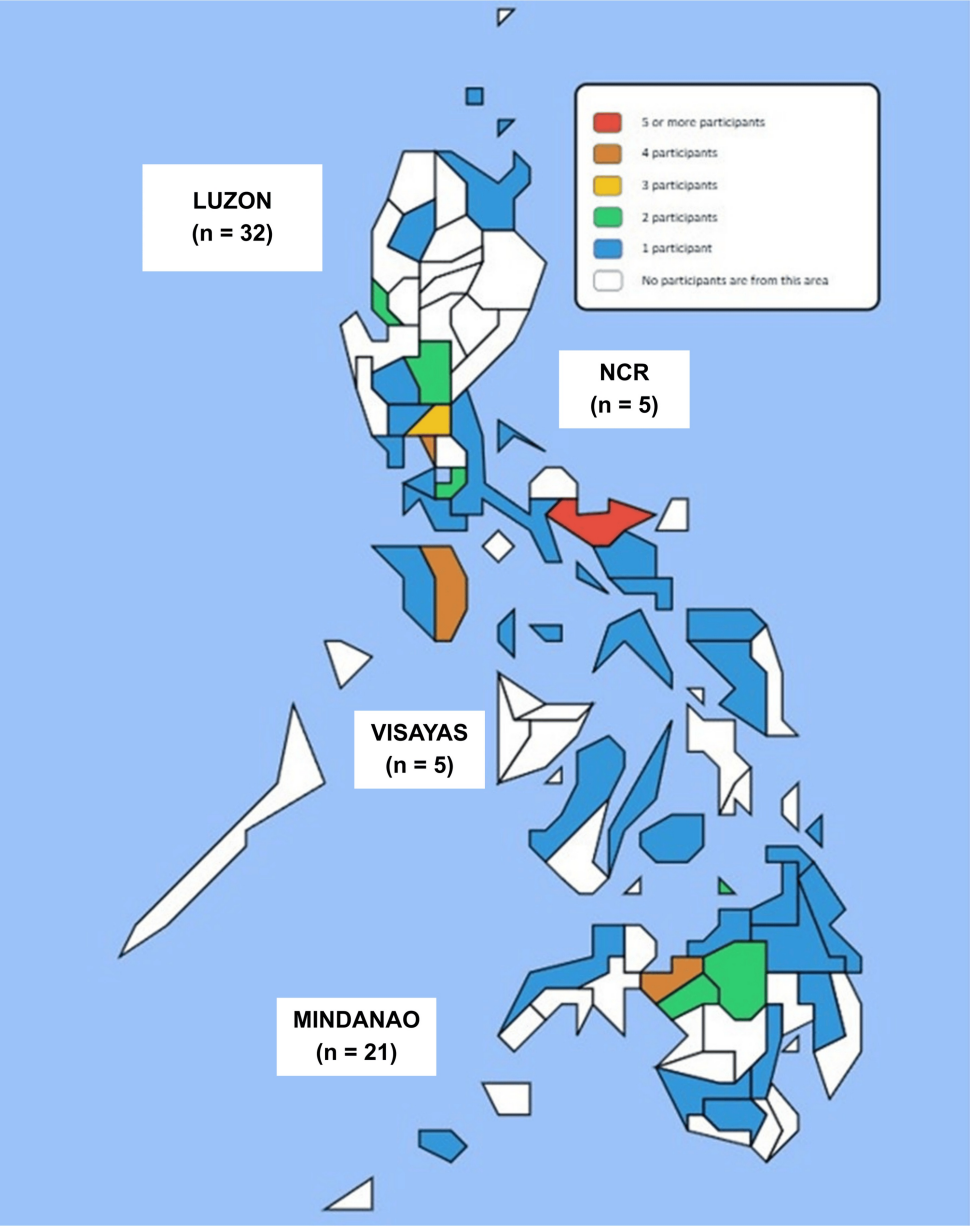
Geographic Distribution of Community Health Physician Participants in the Philippines. Map courtesy of My Philippines Travel Level. NCR: national capital region.

Figure 1 illustrates the distribution of participants across various provinces in the Philippines. In terms of island groups, the majority of participants are from Luzon (32, 50%), followed by Mindanao with 21 (34%) participants. The Visayas accounts for 5 (8%) participants, which is the same percentage as those from the National Capital Region 5 (8%). The majority of participants (44, 69.8%) are employed or previously employed in Rural Health Units under the Department of Health, followed by those with experience working in Barangay Health Stations 19 (30.2%).

The participants in the study are categorized according to the types of municipalities in the Philippines, based on their community's income classification as defined by Republic Act No. 119648. Local Government Units (LGUs) are classified as follows: first-class municipalities have an annual average income of P200,000,000 or more; second-class municipalities earn between P160,000,000 and P200,000,000; third-class municipalities have an income ranging from P130,000,000 to P160,000,000; fourth-class municipalities earn between P90,000,000 and P130,000,000; and fifth-class municipalities have an average annual income of less than P90,000,000. The majority of participants, 26 (41%), are affiliated with first-class municipalities, followed by those from second-class municipalities (11, 17.5%). Additionally, 8 (12.7%) are from third-class municipalities, 10 (15.9%) from fourth-class municipalities, 6 (9.5%) from fifth-class municipalities, and 2 (3%) from sixth-class municipalities.

Although most participants are affiliated with first-class municipalities, 30 (48%) are from GIDA. The Philippine Department of Social Welfare and Development defines GIDA as communities with marginalized populations that are physically and socio-economically separated from mainstream society. These areas are characterized by various physical factors, such as isolation due to distance, and socio-economic challenges, including high poverty rates or the need to recover from crises or armed conflicts [8]. The predominant demographic among participants consists of general physicians (31, 49.2%), followed by family medicine specialists 13 (20.6%).

Management of neurosurgical cases

**Figure 2:**
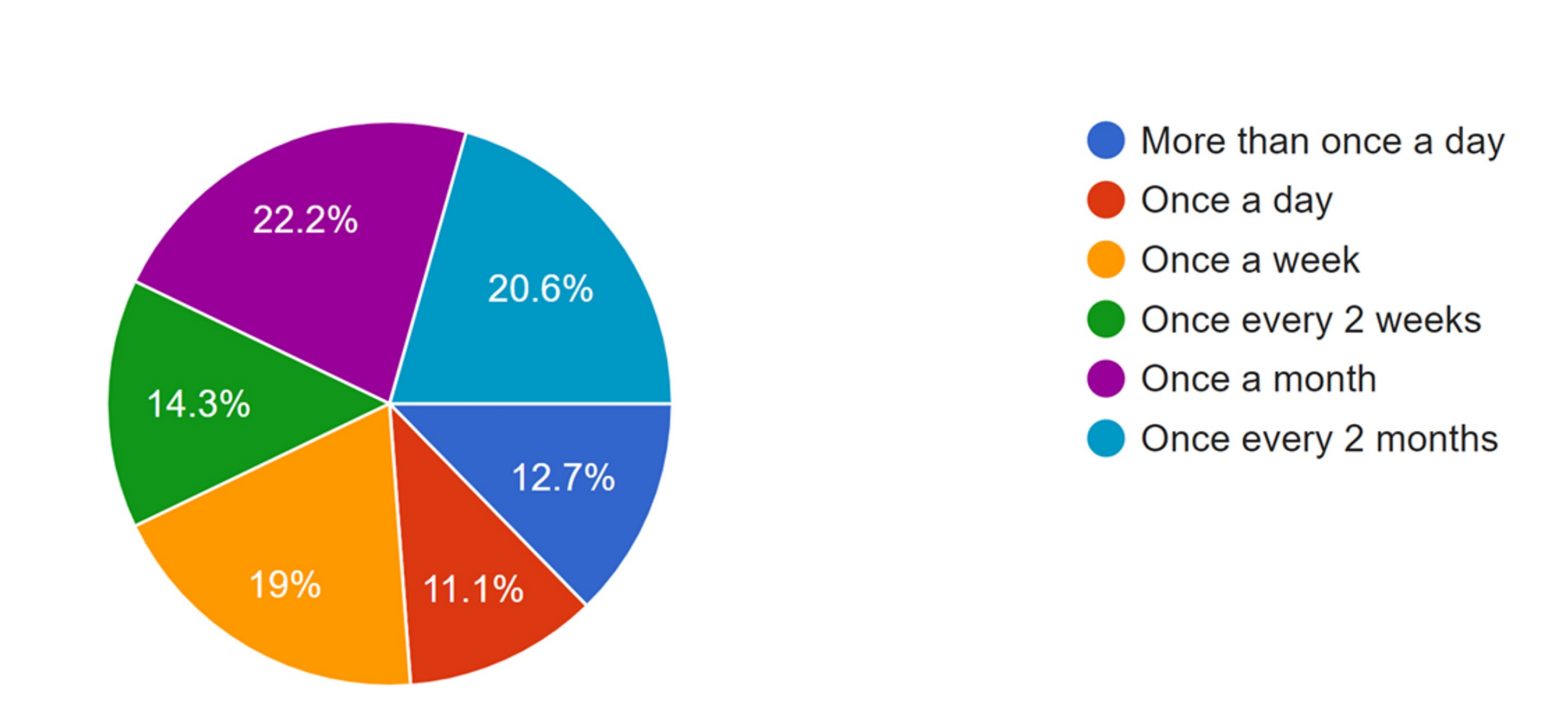
Frequency of Encountering Suspected Neurosurgical Cases among Community Health Physicians. Neurosurgical cases refer to conditions identified at the community level that require initial stabilization and referral, rather than definitive neurosurgical intervention.

As shown in Figure 2, a significant number of physicians report encountering neurosurgical cases at least once a month (22.2%), although the frequency varies across participants.

Notably, a subgroup analysis from Mindanao reveals that 9 (43%) of participants engage in neurosurgical consultations at least once a day. This finding aligns closely with the data from Visayas, where 4 (80%) of participants report seeing neurosurgical cases at least once a week. This is congruent with the data from participants with reported GIDA communities, where 8 (27%) of the participants see a neurosurgical case once a week, and 8 (27%) see a neurosurgical case once a month. In this study, “encountering neurosurgical cases” refers to identifying suspected neurosurgical conditions, providing initial stabilization within the scope of community-based services, and facilitating referral to higher-level facilities, rather than providing definitive neurosurgical procedures.

**Figure 3:**
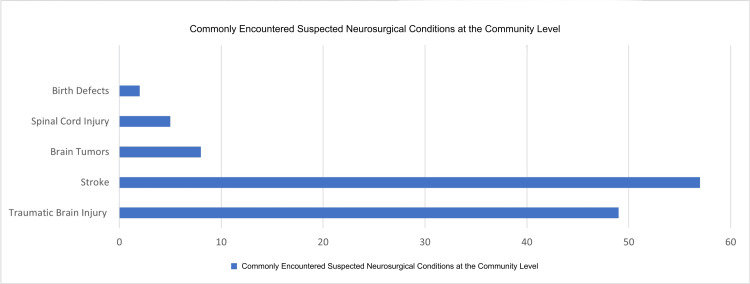
Commonly Encountered Suspected Neurosurgical Conditions Among Community Health Physicians. The conditions shown represent the most frequently identified neurosurgical presentations at the community level that prompted initial assessment, stabilization, and referral. This figure reflects commonly encountered cases included in the survey instrument and is not intended to capture the full spectrum of neurosurgical subspecialties or definitive neurosurgical procedures.

As seen in Figure 3, the predominant neurosurgical consult encountered by community health physicians is stroke. Stroke was reported by 58 (90%) participants, while traumatic brain injury was reported by 50 (79%) participants. Other reported cases are brain tumors, spinal cord injuries, and birth defects. The survey emphasized commonly encountered neurosurgical presentations at the community level and did not aim to capture the full spectrum of tertiary neurosurgical subspecialty case-mix.

The reported capabilities of the community health units affiliated with the participants indicate that a majority are proficient in oxygen supplementation, intravenous access, and wound suturing. Among these units, 17 (27%) are accredited as Primary Health Care facilities by the Department of Health’s Health Facilities and Services Regulatory Bureau. These rural health units are equipped to provide essential primary care services, serving as the initial point of contact for patients and offering coordinated, comprehensive care for a range of presenting conditions, while facilitating referrals to other health delivery systems as needed. These capability data provide context for interpreting referral practices and perceived barriers, particularly regarding stabilization capacity and diagnostic limitations prior to transfer.

**Figure 4:**
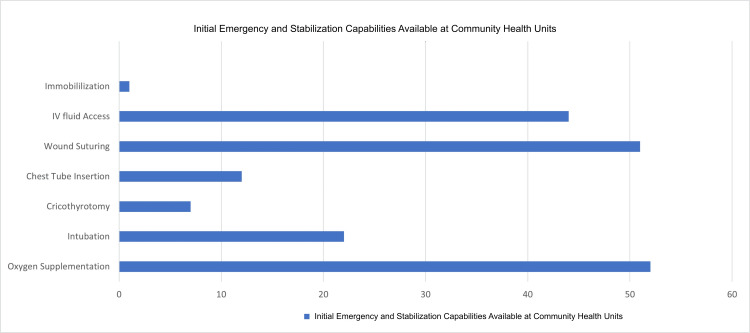
Initial Emergency and Stabilization Capabilities Available at Community Health Units Affiliated with the Participants. These capabilities represent basic emergency measures used for early stabilization of suspected neurosurgical patients prior to referral and do not constitute definitive neurosurgical care. The figure highlights the level of preparedness at the first point of contact in the health system and contextualizes referral delays and downstream barriers described by the participants.

As shown in Figure 4, when the participants were asked how they cope with the significant limitations in managing neurosurgical cases, the primary challenge they noted is the need for timely referrals to tertiary hospitals, which can be located anywhere from a 20-minute drive to six hours away. Many patients must first be sent to district hospitals or private institutions due to the lack of essential diagnostic tools at rural health units (RHUs), resulting in delays in care. Furthermore, local government units often cannot provide basic diagnostic resources, necessitating additional referrals to Level 1 hospitals for laboratory procedures. Participants reported that they typically provide initial care, such as pressure dressings and IV lines, before referring patients for more advanced management. This situation is compounded by limited access to medications and diagnostic facilities, which restricts effective treatment at the community level. Diagnostic tests often require visits to private laboratories that operate only during business hours, delaying necessary interventions outside those times. Participants commonly suggested the need for improved funding for healthcare resources, more efficient referral pathways, and stronger coordination with nearby facilities to improve access to diagnostics and timely care.

Referral systems

**Figure 5:**
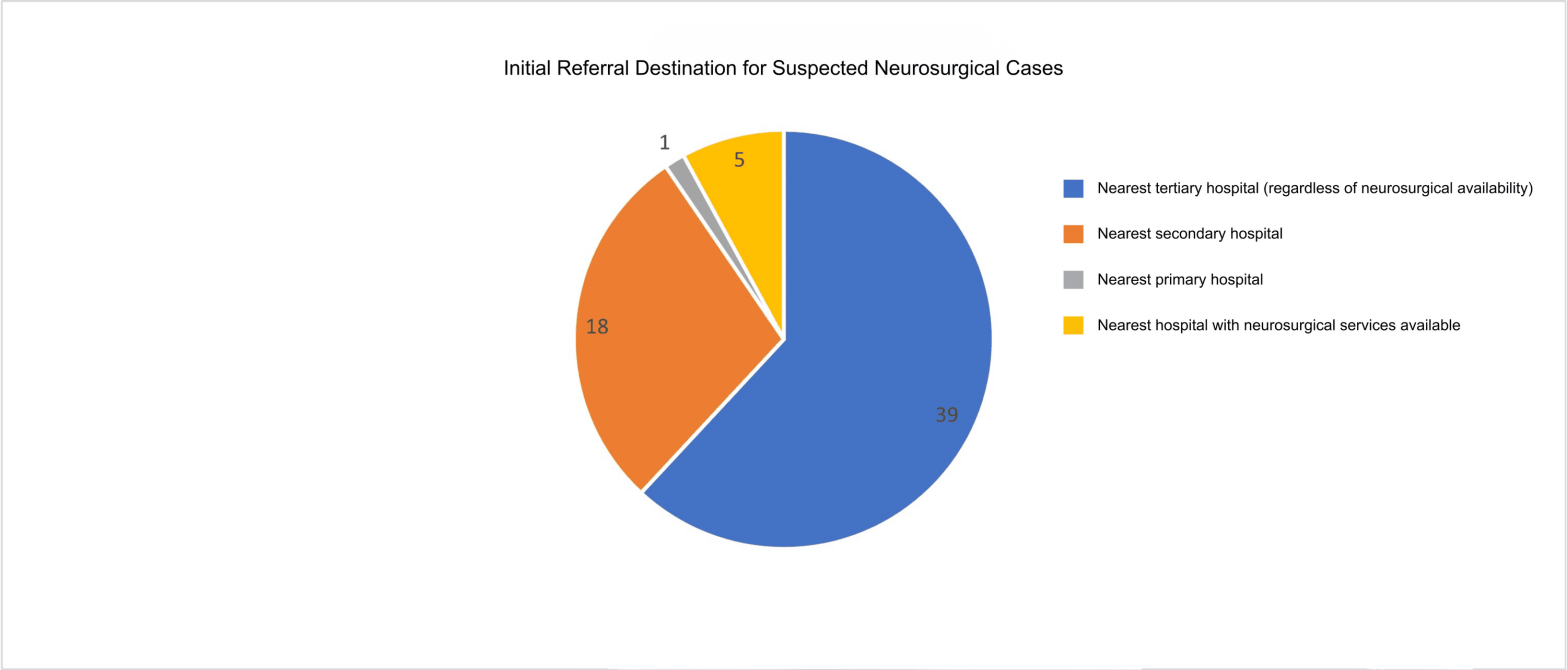
Initial Referral Destinations for Suspected Neurosurgical Cases as Reported by Community Health Physicians. Referral choices reflect proximity, transport feasibility, and institutional accessibility rather than guaranteed availability of neurosurgical services. In many settings, secondary or non-neurosurgical tertiary hospitals serve as intermediary referral points prior to transfer to definitive neurosurgical centers.

Figure 5 illustrates the referral patterns for neurosurgical patients as reported by the participants. A significant majority, comprising 39 (61.9%) participants, indicated that they refer patients to the nearest tertiary hospital.

Only 11 (17%) direct referrals to hospitals accredited by the Philippine Board of Neurosurgery. Notably, 40 (63%) refer patients to institutions accredited by the Philippine Board of General Surgery, ensuring that a consultant neurosurgeon is available at these facilities [9].

**Figure 6:**
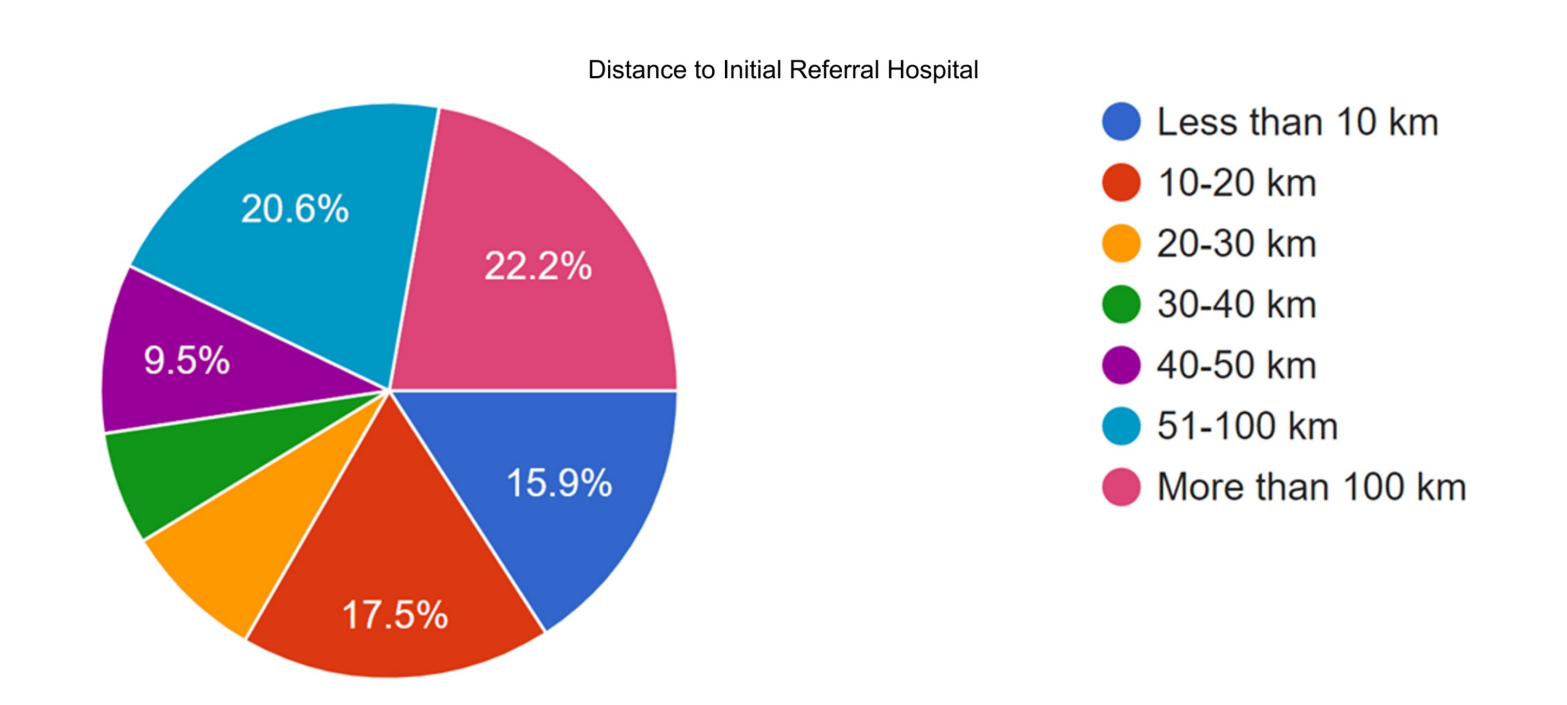
Reported Distance Between the Community and the Initial Referral Hospital for Suspected Neurosurgical Cases. Distances represent estimated travel length from the community health unit to the first referral facility and do not distinguish between inter-island and intra-island transfers. Reported distances reflect perceived access rather than actual transport time, which may vary depending on terrain, availability of transport, and weather conditions, particularly in geographically isolated and disadvantaged areas.

Figure 6 illustrates the distance to preferred referral hospitals. Fourteen participants (22.2%) reported that their community is situated more than 100 kilometres from the nearest hospital, while thirteen participants (20.6%) indicated distances between 51 and 100 kilometres. Among GIDA communities, 12 (40%) are more than 100 kilometres away, and 10 (33.3%) are 51-100 kilometres away. 

**Figure 7:**
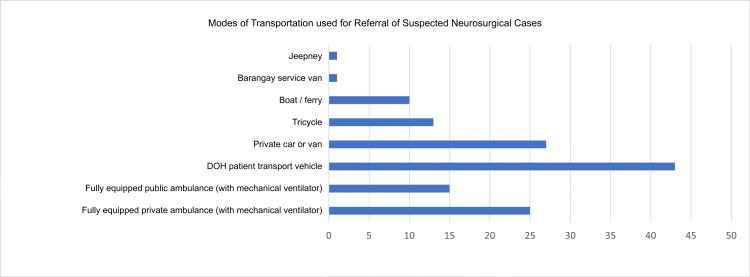
Modes of Transportation Used for Referral of Suspected Neurosurgical Cases from Community Health Units to Referral Hospitals. Both formal (Department of Health transport vehicles and ambulances) and informal modes of transport (private vehicles, boats, tricycles, and jeepneys) were reported. The presence of non-medical transport highlights potential delays and limitations in prehospital stabilization, particularly for patients requiring urgent neurosurgical care.

As shown in Figure 7, regarding transportation modes utilized by the community, a majority of 42 communities have access to a patient transport vehicle provided by the Department of Health. Notably, 10 communities also benefit from boat or ferry services, reflecting the archipelagic nature of certain areas that necessitate water transport. However, only 25 communities have access to fully equipped public ambulances with mechanical ventilators, which represents the ideal setup for patient transport. Additionally, 27 communities continue to rely on private cars or vans to transport patients to their preferred referral hospitals.

In terms of the transport time to the preferred referral hospital, a majority of participants (30, 47%) reported transport times of less than one hour, followed by 22 (35%) reporting transport times of one to three hours. A substantial (47, 74.6%) of participants indicated that they would provide patients with a referral form from the community health unit to present at the nearest treatment facility. Additionally, 42 (66.7%) expressed their willingness to make direct phone calls to the facility on behalf of the patients, while only 9 (15%) would choose to contact the referring facility via SMS. One (1.6%) participant has noted that he sends an email referral through the DOH referral system.

Local government support

The participants were surveyed about their perceptions of the government's role in financing healthcare for neurosurgical cases. Notably, 25 (39.7%) reported receiving a satisfactory level of support from local authorities. When asked about the specific types of assistance provided, 57 (90.5%) participants indicated that they primarily benefit from transportation support from the local government. Additionally, 33 (52.4%) participants mentioned that their patients receive funding for hospitalization from local government resources.

Regarding training, 44 (69.8%) of participants indicated that they did not receive any training in the management of trauma or neurosurgical patients. Only 6 (9.5%) reported receiving training once or more than once a year. Participants were also asked about the follow-up system for patients who have undergone neurosurgical procedures. A significant majority (50, 79.4%) noted that there is no follow-up system in place for these patients, leading them to either remove sutures themselves or forgo proper post-operative care.

A notable approach involves the use of endorsement or referral forms sent back to the rural health unit (RHU) detailing the patient's diagnosis, medications, and follow-up schedule. After stabilizing a neurosurgical patient, healthcare providers often refer them to the nearest tertiary or secondary hospital, accompanied by necessary details for the receiving facility. However, it was noted that there is typically no downward referral from hospitals back to RHUs, leading to a system primarily focused on upward referrals. Patients are reminded about their follow-up appointments at tertiary hospitals. Some providers also mentioned home visits after hospital discharge, though follow-ups often occur at the original institution. Overall, there appears to be a structured yet limited process in place for ensuring proper follow-up care for neurosurgical patients within the community health system.

Barriers and challenges

Participants identified financial constraints as the primary barrier to accessing neurosurgical care within their communities, followed by logistical challenges and geographical isolation. These obstacles compromise the quality of care that can be provided, limiting access to essential resources that should be available to patients. Participants also highlighted shortages in human resources and limited training opportunities related to neurosurgical and trauma case management. Furthermore, participants noted gaps in infrastructure needed for the ongoing care of neurosurgical patients. Nineteen participants (30.2%) reported reliance on traditional healers within their communities, which may hinder timely referral to higher-level medical facilities. The questionnaire did not systematically capture specific cultural practices beyond this reported reliance, and this is addressed as a limitation.

Community health physicians provided recommendations to enhance access to neurosurgical services. They emphasized the need for a better referral system and additional public tertiary hospitals that could cater for neurosurgical cases. Participants also noted the importance of basic training for medical staff, improved logistical support, and resource allocation to enable quality first-aid care and rapid diagnostics. They highlighted the role of political support and community engagement, including research initiatives to inform LGUs and the public. Additional recommendations included strengthening training for healthcare workers in basic neurosurgical care and management, developing trauma centres, improving equipment availability, enhancing public-private partnerships, and implementing community education on neurosurgical emergencies and lifestyle modification. These recommendations reflect participant-reported priorities and are presented descriptively as part of the survey findings.

## Discussion

Access to essential neurosurgical care in low-resource settings remains constrained by multiple interrelated barriers. In this study, community health physicians identified financial, logistical, geographic, and workforce-related challenges as perceived barriers to timely neurosurgical referral and care within their respective communities. These findings reflect frontline perspectives rather than direct clinical outcomes and should be interpreted within the descriptive and exploratory scope of the study [3].

Financial constraints emerged as the most frequently reported barrier. Globally, approximately 3.7 billion individuals are at risk of catastrophic health expenditure when surgical care is required, with the burden disproportionately affecting low and middle-income countries (LMICs) [10]. Participants in this study reported that out-of-pocket costs related to diagnostics, transportation, and hospitalization often delayed referral decisions or limited access altogether. While this study did not measure patient-level outcomes, existing literature suggests that financial barriers may contribute to delays in care, which are associated with poorer neurological outcomes in time-sensitive conditions such as stroke and traumatic brain injury [11,12].

Limited availability of neurosurgical services remains a persistent challenge across LMICs. Prior studies have demonstrated substantial disparities in neurosurgeon density, with lower-income countries reporting significantly fewer specialists per population compared with high-income settings [13,14]. For example, sub-Saharan African countries such as Uganda, Tanzania, and Ethiopia report neurosurgeon densities far below global averages, while middle-income countries in South and Southeast Asia continue to experience uneven regional distribution of services. These international patterns provide context for the Philippine experience described by participants, without implying uniform conditions across all LMICs or regions.

Geographic isolation and transportation constraints were also commonly reported. Participants described long travel distances, inter-island transfers, and limited availability of equipped ambulances, particularly in geographically isolated and disadvantaged areas (GIDAs). Although this study did not assess treatment timelines or clinical endpoints, prior literature indicates that delays in access to neurosurgical evaluation and intervention may be associated with increased morbidity and mortality, particularly in emergency cases [13]. The Philippine archipelagic geography likely amplifies these challenges, mirroring patterns observed in other island LMICs.

Workforce and training gaps were another major concern. A large proportion of respondents reported limited or no formal training in trauma or neurosurgical emergency management. This finding reflects perceived preparedness rather than measured competency but aligns with reports from other middle-income countries where community-based physicians express the need for structured neurosurgical emergency training [15-17]. Addressing these gaps through targeted educational initiatives may improve early recognition, stabilization, and referral processes, although the impact on patient outcomes requires further study.

Participants also emphasized the absence of structured follow-up and downward referral mechanisms. Most reported that neurosurgical patients were referred upward to higher-level facilities without systematic feedback to community health units after discharge. While this study did not track post-referral outcomes, the lack of continuity of care was perceived to hinder long-term management and rehabilitation. Strengthening bidirectional referral systems may improve coordination between tertiary hospitals and community-based providers.

Cultural factors, including reliance on traditional healers, were reported by some participants as influencing care-seeking behavior. These observations highlight the importance of community engagement and culturally sensitive health education to promote timely referral and trust in formal health systems.

Actionable recommendations and future directions

Based on participant perspectives, several actionable strategies may be considered as: development of standardized referral and feedback protocols between tertiary hospitals and community health units, expansion of short-course training programs on neurosurgical emergencies for community health physicians, strengthening public-private partnerships to improve access to diagnostics and transportation, and integration of community education programs focused on early recognition of neurosurgical emergencies.

Future research should adopt multimodal and multi-institutional approaches, including collaboration with the Department of Health to improve sampling representativeness. Nationwide inventories of neurosurgical infrastructure, equipment, neuroanesthesia services, intensive care capacity, and specialized personnel would provide a more comprehensive assessment of neurosurgical readiness. Prospective referral-mapping studies examining inter-regional transfer patterns, transportation modalities, time-to-care, and patient outcomes would further strengthen understanding of access barriers and system performance.

Study limitations

This study has several limitations that should be acknowledged. First, the use of snowball sampling and online data collection introduces potential selection bias and may underrepresent community health physicians practising in areas with limited internet or technological access, particularly in remote GIDA communities. Second, the study intentionally focused on public and community-based healthcare settings, excluding private-sector practice, which may limit generalizability to the entire neurosurgical care landscape. Third, data were self-reported, raising the possibility of recall and reporting bias. Fourth, the study did not collect patient-level clinical outcomes, such as morbidity, mortality, or treatment success, and therefore cannot establish causal relationships between identified barriers and health outcomes. Finally, the exploratory design limits nationwide generalization, and findings should be interpreted as reflecting the perceptions of participating physicians rather than definitive system-wide performance metrics.

## Conclusions

This study highlights three primary challenges to neurosurgical access in the Philippine community health setting as perceived by community health physicians: financial constraints, logistical and geographic barriers to referral, and limited training in neurosurgical case management. These issues were consistently identified across regions, including geographically isolated and disadvantaged areas, underscoring systemic gaps in access to specialized surgical care.

Based on participant responses, the most critical needs include strengthening referral systems to reduce delays, improving transportation and diagnostic support at the community level, and enhancing training opportunities for community health physicians in the initial recognition and stabilization of neurosurgical emergencies. Participants also emphasized the importance of improving coordination between community health units and tertiary hospitals to support continuity of care. While the findings reflect frontline perspectives rather than clinical outcomes, they provide valuable insight into barriers encountered at the first point of patient contact. Addressing these challenges through targeted policy interventions, capacity-building initiatives, and system-level coordination may contribute to more equitable access to neurosurgical care, particularly for underserved and geographically isolated populations in the Philippines.

## Appendices

Appendix A

**Table 1:** Survey Questionnaire. RHU: rural health unit; BHS: Barangay health station; GIDA: geographically isolated and disadvantaged area; DOH: Department of Health; PT/APTT: Prothrombin time/activated partial thromboplastin time.

Domain	Item	Response Options
Section 1: Demographics and Professional Background	1. Full name (optional)	
	Position/Title	
	2. Medical specialty	General Medicine; Family Medicine; Emergency Medicine; Surgery; Pediatrics; Internal Medicine; Other
	3. How many years have you practised as a physician?	<5; 5–10; 11–20; >20 years
	4. Are you currently working as a Public Health Officer?	Yes/No
	5. If you answered “No,” how many years ago did you work as a Public Health Officer?	<5; 5–10; 11–20; >20 years
	6. Where was your current or previous location of community practice? Region	
	Municipality	
	Community/Barangay	
	7. Types of facilities where you have had community practice (check all that apply):	RHU; BHS; City Health Office; Local Government Hospital; Private Clinic; Other
	8. Income classification of your municipality (average annual income):	(PHP-based categories) First to Sixth Class (55,000 000 to <15,000,000)
	9. Does your municipality belong to a Geographically Isolated and Disadvantaged Area (GIDA)?	Yes/No
Section 2: Management of Neurosurgical Cases	10. How often do you encounter neurosurgical cases, including trauma, in your practice?	>Once a day; Once a day; Once a week; Once every two weeks; Once a Month; Once every 2 months
	11. What types of neurosurgical conditions do you most commonly see? (Check all that apply)	TBI; Stroke; Brain tumors; Spinal cord injury; Myelomeningocele; Other
	12.What is the capability of your unit in providing emergency surgical care? (Check all that apply)	Oxygen; Intubation; Cricothyrotomy; Tracheostomy; Chest tube; Suturing; IV access; Other
	13. What diagnostic tools are available in your facility? (Check all that apply)	X-ray; CT; Ultrasound; CBC; Electrolytes; Cougulation studies (PT/APTT); Urinalysis
	14. If you do not have access to these diagnostic tools, how do you manage this limitation?	
Section 3: Referral Process	15. How do you typically refer patients needing neurosurgical care?	Secondary hospital; Tertiary hospital; Specialised centre; Other
	16. What is the average distance from your facility to the nearest tertiary hospital?	<10 km; 10-20 km; 20-30 km; 30-40 km; 40-50 km; 51-100 km; >100 km
	17. Which hospitals do you send your neurological patients to?	
	18. What transportation options are available for patients travelling to referral hospitals? (Check all that apply)	Public ambulance; Private ambulance; DOH transport; Private vehicle; Tricycle; Boat; Other
	19. How long does it typically take for a patient to travel from your facility to the nearest referral centre?	<1 hour; 1-3 hours; 4-6 hours; >6 hours
	20. How do you refer your patient to the nearest neurosurgical-capable centre?	Phone; SMS; Referral form; Email
Section 4: Local Government Support	21. How would you rate the level of support from the local government in terms of health financing for neurosurgical cases?	Excellent; Good; Fair; Poor; Very poor
	22. What type of logistical support is provided by the local government? (Check all that apply)	Referral funding; Transport; Supplies; Training; Other
	23. How often do you receive training for the management of trauma or neurosurgical patients?	>Once/year; Every 2 years; Every 5 years; None
	24. Is there a follow-up system in place for patients who have undergone neurosurgical procedures?	Yes/No
	25. If yes, please describe the follow-up procedures	
Section 5: Barriers and Challenges	26. What are the main barriers to accessing neurosurgical care in your area? (Check all that apply)	Geographic; Cultural; Technical; Logistical; Technological; Financial; Other
	27. How do these barriers affect the quality of care provided? (Check all that apply)	Training gaps; Communication gaps; HR shortages; Infrastructure limitations; Traditional beliefs; Policy inconsistency; Other
	28. What improvements or changes would you suggest to enhance neurosurgical care in your communities?	
Section 6: Additional Comments	29. Do you have any additional comments or suggestions regarding neurosurgical care in your community?	
